# 3D residual attention hierarchical fusion for real-time detection of the prostate capsule

**DOI:** 10.1186/s12880-024-01336-y

**Published:** 2024-06-24

**Authors:** Shixiao Wu, Chengcheng Guo, Ayixiamu Litifu, Zhiwei Wang

**Affiliations:** 1 School of Information Engineering, Wuhan Business University, 816 Dongfeng Avenue, Caidian District, Wuhan, Hubei 430056 China; 2 School of Electronic Information, Wuhan University, 129 Luoyu Road, Hongshan District, Wuhan, Hubei 430072 China; 3 School of Physics and Electronic Information, Xinjiang Normal University, Urumqi, China; 4 Department of Cardiothoracic Surgery, People’s Hospital of Wuhan University, 99 Zhangzhidong Road, Wuchang District, Wuhan, Hubei 430060 China

**Keywords:** SimAM, Residual attention hierarchical fusion, Deep learning, Machine learning, Prostate capsule

## Abstract

**Background:**

For prostate electrosurgery, where real-time surveillance screens are relied upon for operations, manual identification of the prostate capsule remains the primary method. With the need for rapid and accurate detection becoming increasingly urgent, we set out to develop a deep learning approach for detecting the prostate capsule using endoscopic optical images.

**Methods:**

Our method involves utilizing the Simple, Parameter-Free Attention Module(SimAM) residual attention fusion module to enhance the extraction of texture and detail information, enabling better feature extraction capabilities. This enhanced detail information is then hierarchically transferred from lower to higher levels to aid in the extraction of semantic information. By employing a forward feature-by-feature hierarchical fusion network based on the 3D residual attention mechanism, we have proposed an improved single-shot multibox detector model.

**Results:**

Our proposed model achieves a detection precision of 83.12% and a speed of 0.014 ms on NVIDIA RTX 2060, demonstrating its effectiveness in rapid detection. Furthermore, when compared to various existing methods including Faster Region-based Convolutional Neural Network (Faster R-CNN), Single Shot Multibox Detector (SSD), EfficientDet and others, our method Attention based Feature Fusion Single Shot Multibox Detector (AFFSSD) stands out with the highest mean Average Precision (mAP) and faster speed, ranking only below You Only Look Once version 7 (YOLOv7).

**Conclusions:**

This network excels in extracting regional features from images while retaining the spatial structure, facilitating the rapid detection of medical images.

## Introduction

A major focus of computer vision research is improving feature representations and precisely capturing important object features in images. According to recent developments, networks can capture spatial feature correlations more efficiently when learning mechanisms are integrated into them. Convolutional neural networks’ feature extraction capabilities are improved by this integration. Effective strategies to improve feature extraction skills have been found, including feature fusion and the incorporation of attention mechanisms [1, 2].

Jianhui Yu et al. proposed an attention-based convolutional neural network model specifically for medical point clouds, namely 3D Medical Point Converter (3DMedPT), for detecting complex biological structures [3]. Tianyu Shi et al. proposed a novel network for segmenting acute ischemic stroke (AIS) lesions from four computed tomography (CT) perfusion images [4]. Their approach is built on the idea that incorporating cross-modal and cross-attention mechanisms can be advantageous for this task. Duran et al. proposed a new end-to-end multi-class network for co-segmenting prostate and cancer lesions by gleason score (GS) group grading [5]. A novel multimodal multi-head convolutional attention module for super-resolution CT and magnetic resonance imaging (MRI) scanning was proposed by Georgescu et al. [6]. Building upon traditional convolutional neural networks, Furui Bai and colleagues enhanced the model by integrating a convolutional attention mechanism, which leverages weighted jump connections [7]. In a separate development, Yusuke Takagi and his team introduced a Personalized Attention Mechanism designed to dynamically adjust the focus areas within medical images, taking into account associated clinical records [8]. This approach notably utilizes a modified transformer architecture to map the intricate interplay between medical imagery and textual clinical data. Further, Jianfang Wu and his team developed a novel approach for classifying diabetic retinopathy, employing a technique centered on visual transformation [9].

Danni Ai et al. introduced a rapid multi-scale fusion algorithm for heartbeat classification, comprising four key stages: pre-processing, feature extraction, feature fusion, and classification. The feature fusion approach utilizes the tension-based multi-line subspace learning method [10]. Xinsheng Zhan proposed a multiple feature fusion mechanism for micro-calcified clusters in X-ray images, involving double sampling on the underlying feature map followed by horizontal connection to the previous layer [11]. Atkale et al. suggested a multi-scale feature fusion model for facial aging, featuring 5 parallel branches and employing up-sampling and down-sampling operations through pooling, convolution, and cavity convolution [12]. Bakkouri et al. presented a 3D multi-scale feature fusion algorithm with four levels, each comprising four 3D-CNN branches of identical architecture but different parameters [13]. The integration of multi-scale features in feature fusion requires considerations such as spatial dimension consistency and the merging and selection of feature maps. While these algorithms address these aspects, they may overlook the crucial balance between network precision and speed post feature fusion.

Moreover, the latest state-of-the art object detection technologies designed for natural images may not always be suitable for heterogeneous medical images with significant scale variations and complex backgrounds. Faster speed and higher precision can improve the safety and success rate of surgery, reducing the probability of surgical complications such as capsule perforation. This paper addresses the challenge of rapidly detecting the prostate capsule by developing a detection network that integrates a 3D attention-free residual network and progressive fusion of forward features.

## Methods

### Attention-based single shot multibox (ASSD)

**Fig. 1:**
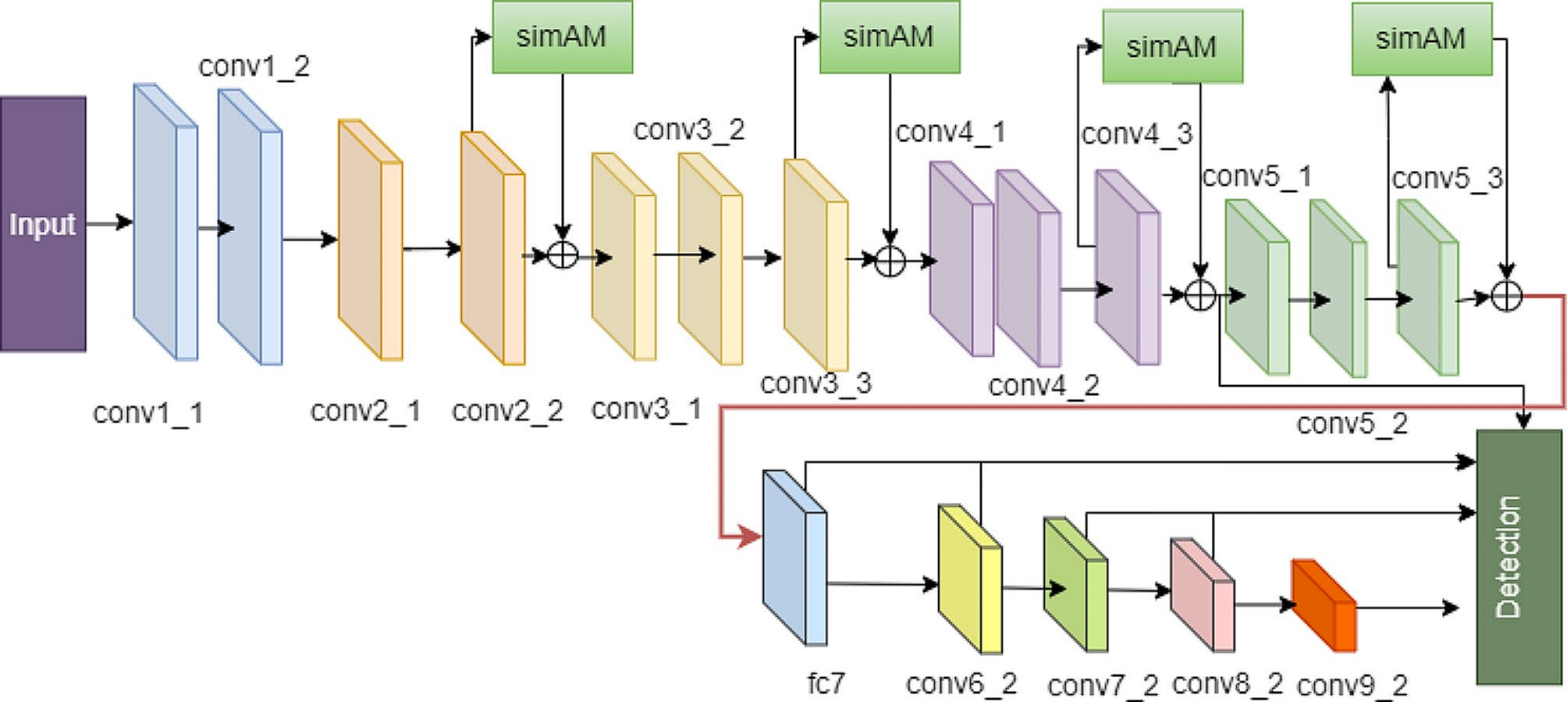
The ASSD network

We apply SimAM to augment the features of four convolutional layers in VGG16 (Fig. 1). SimAM is a parameterless attention mechanism, and incorporating its 3D attention module ensures the network’s speed in ASSD.

Building upon VGG16, ASSD enhances conv2_2, conv3_3, conv4_3, conv5_3, etc., through attention-based mechanisms, thereby enhancing low-level feature extraction capabilities. After the convolution layer, four modules are incorporated, with BatchNorm and Rectified Linear Units(ReLU) layers aiding in network convergence acceleration. BatchNorm, a technique aimed at stabilizing input distribution within layers, allows for control over mean and variance through an additional network layer. This enables the model to utilize a broader spectrum of learning rates and facilitates faster convergence during the training process.

### Multi-scale feature fusion single shot multibox detector (MFFSSD)

**Fig. 2:**
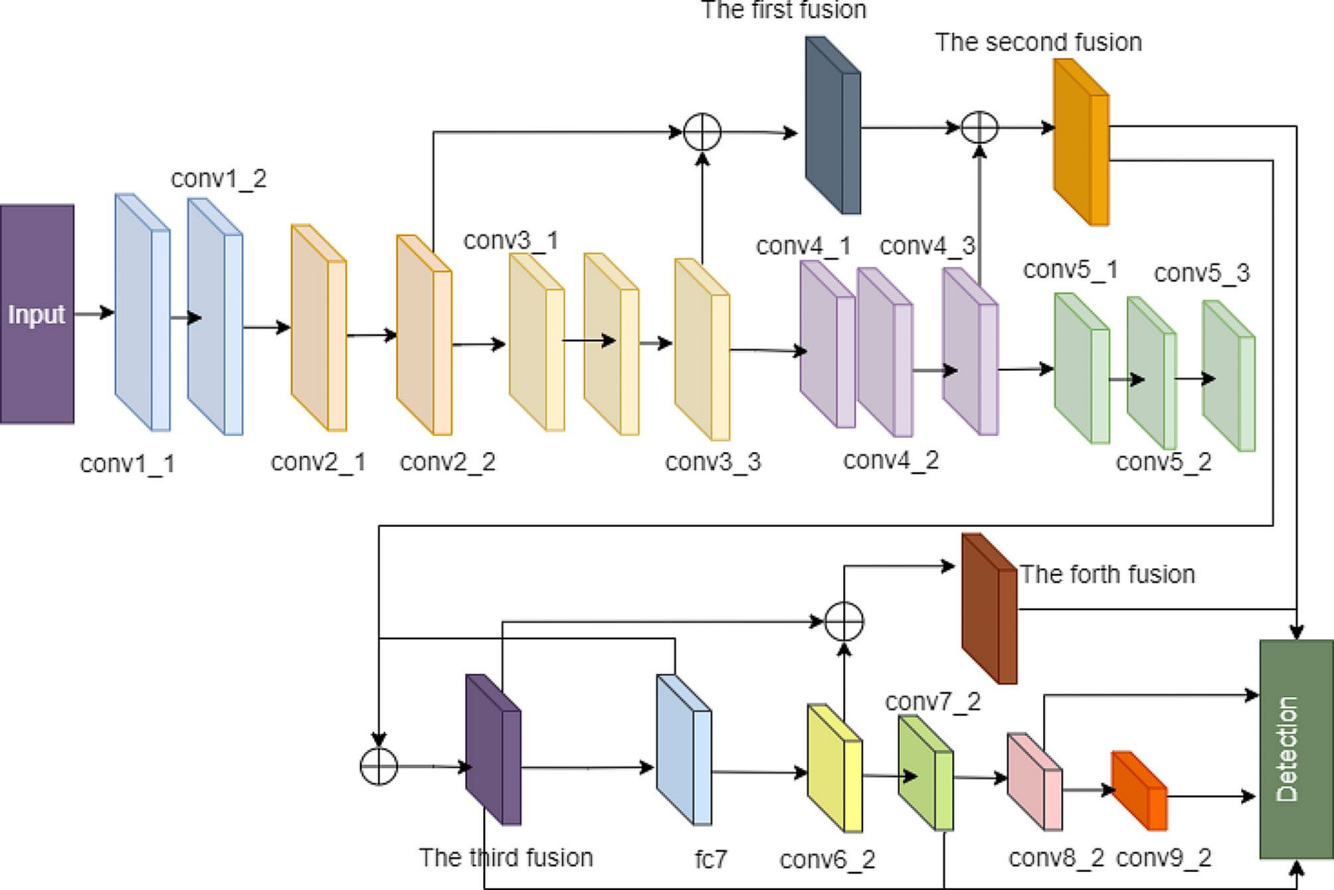
The MFFSSD network

To enable the network to learn more discriminative neurons, it is essential to consider spatial and channel dimensions while allowing for flexible attention weight adjustments. By incorporating a three-dimensional attention fusion of features using an attention-free mechanism, the network can extract features more effectively. Additionally, relying on a shallow network can weaken the generalization ability and result in lower detection precision. Hence, the feature fusion network Multi-scale Feature Fusion Single Shot Multibox Detector is employed to increase the network’s depth and enhance its feature extraction capabilities. The architecture of the MFFSSD network in AFSSD is illustrated in Fig. 2.

**Fig. 3:**
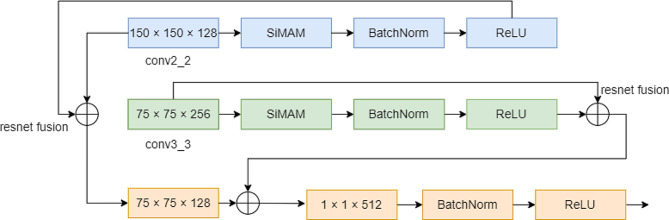
Forward feature stepwise Fusion module (The first feature fusion module)

The MFFSSD module consists of four forward feature stepwise fusion modules, and one of the fusion processes is shown in Fig. 3. In Fig. 3, the first feature fusion module is illustrated. The SimAM module was introduced to conv2_2 (150 × 150 × 128) for enhanced convergence and activation through BatchNorm and ReLU. Subsequently, a residual attention fusion was conducted with the original conv2_2, resulting in a fused feature map size of 75 × 75 × 128. The fusion continued with conv3_3 (75 × 75 × 256) after applying the SimAM module, followed by BatchNorm and ReLU for accelerated convergence and activation. The resultant fused feature map was concatenated with a 75 × 75 × 128 feature map, underwent a 1 × 1 × 512 linear transformation, and further optimized for convergence and activation by BatchNorm and ReLU, leading to a final size of 75 × 75 × 512.

**Fig. 4:**
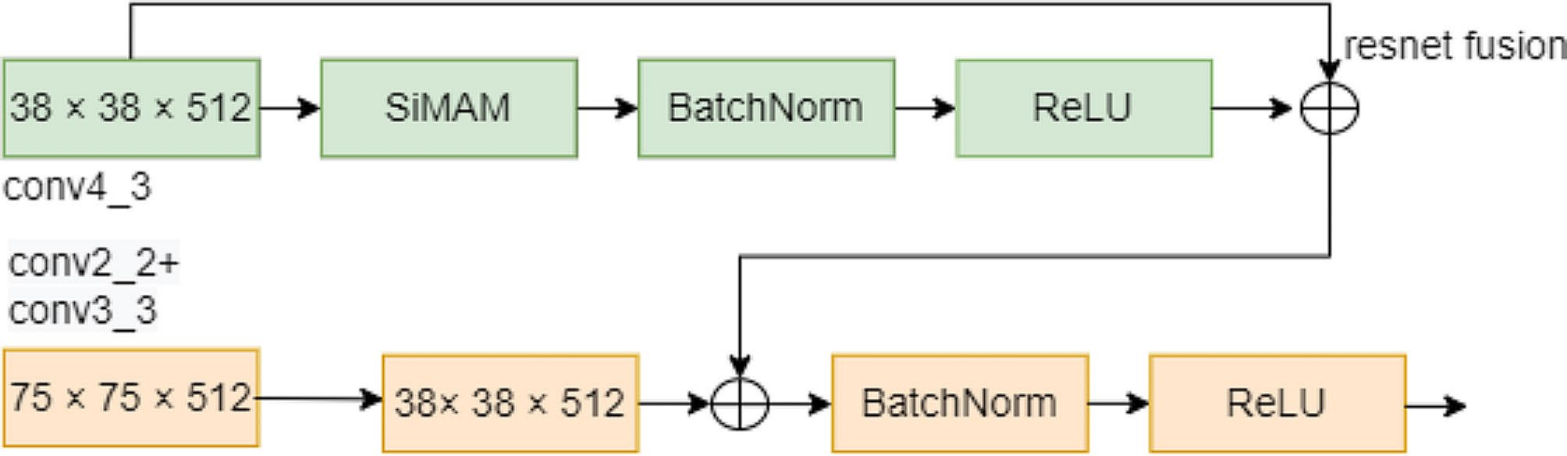
Forward feature stepwise Fusion module (The second feature fusion module)

Fig. 4 showcases the second feature fusion module. Following the integration of the SimAM module, conv4_3 (38 × 38 × 512) underwent acceleration for convergence and activation through BatchNorm and ReLU, and was subjected to residual attention fusion with the original conv4_3. The downsampled first feature fusion module was then merged with the conv4_3 residual attention feature fusion module, with convergence optimization facilitated by BatchNorm and activation by ReLU. Upon completion of the second feature fusion, the resulting size was 38 × 38 × 512.

**Fig. 5:**
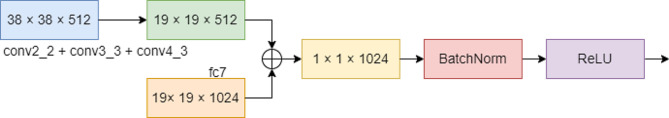
Forward feature stepwise Fusion module (The third feature fusion module)

In Fig. 5, the third feature fusion module is depicted. This module performs double downsampling of the feature map, reducing its size to 19 × 19 × 512. The downsized feature map is then fused with the convolutional layer fc7, resulting in a fused feature map with 1536 channels. Subsequently, a 1 × 1 convolution operation is applied to enhance nonlinearity after fusion, adjusting the number of channels in the feature map to 1024. The adjusted feature map, now sized 19 × 19 × 1024, undergoes convergence acceleration through BatchNorm and activation via ReLU.

After the third feature fusion is completed within this module, the resulting size of the feature map remains consistent at 19 × 19 × 1024. This process ensures that the features are effectively fused and optimized for subsequent stages of the network, maintaining the integrity and quality of the information encoded within the feature maps.

**Fig. 6:**
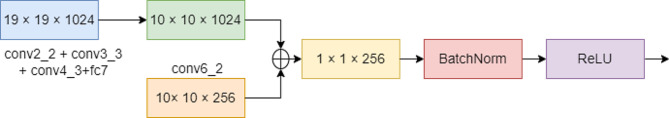
Forward feature stepwise Fusion module (The forth feature fusion module)

In Fig. 6, the fusion process of the fourth feature fusion module is illustrated. The third feature fusion module initiates by performing double downsampling of the feature map, reducing its size to 10 × 10 × 1024. The downsized feature map is then fused with the convolution layer conv6_2, resulting in a fused feature map with 1280 channels. Following this fusion, a 1 × 1 convolution operation is applied to enhance nonlinearity, adjusting the number of channels in the feature map to 256.

The adjusted feature map, now sized at 10 × 10 × 256, undergoes convergence acceleration through BatchNorm and activation via ReLU. Upon completion of the fourth feature fusion process, the resulting size of the feature map is maintained at 10 × 10 × 256. This meticulous fusion and optimization process ensures that the features are effectively integrated and refined for subsequent stages in the network, preserving the quality and integrity of the information encoded within the feature maps.

### AFFSSD

**Fig. 7:**
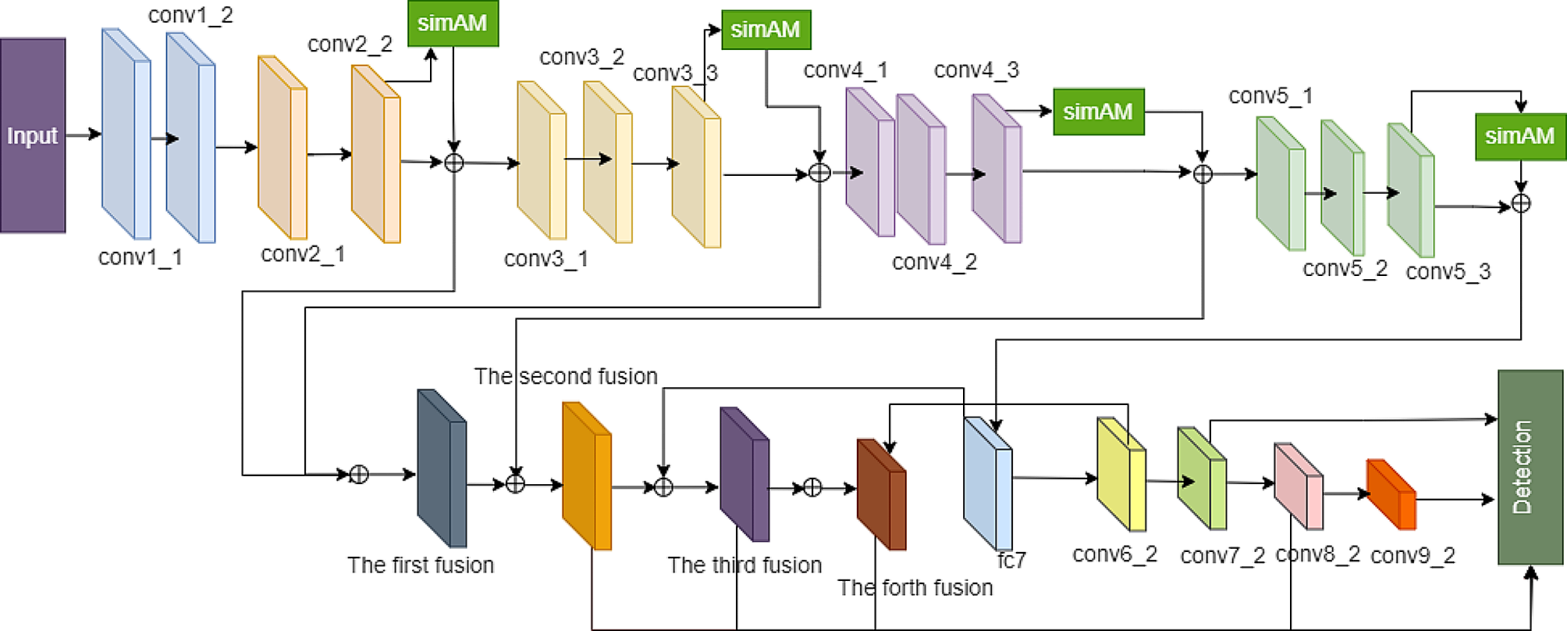
The AFFSSD network

A new model named AFFSSD has been proposed, drawing inspiration from the residual attention fusion model ASSD and the progressive forward feature fusion model MFFSSD. The AFFSSD model combines the progressive fusion of residual attention and forward features, as illustrated in Fig. 7.

SimAM, a component of AFFSSD, dynamically adjusts weights based on the significance of location information, focusing on learning crucial features. This adaptive learning mechanism facilitates rapid network convergence and enhances the independent learning capacity of AFFSSD. Additionally, the four progressive fusion modules of forward features in AFFSSD allow for higher-level convolutions, enabling the extraction of more coarse-grained position information.

By leveraging semantic feature learning, the AFFSSD model can adeptly learn and represent robust spatial position information. This proficiency in capturing spatial details proves beneficial for the accurate classification and precise localization of the prostate capsule. The fusion of attention-based mechanisms and progressive feature fusion in AFFSSD contributes to its superior performance in object detection tasks, particularly in scenarios where precise localization and classification are essential.

## Results

This paper not only compares the AFFSSD model with the SSD model but also delves into the distinctions between the AFFSSD model and other two-stage models like Faster R-CNN, Region-based Fully Convolutional Networks (R-FCN), Sparse R-CNN, as well as one-stage object detection models such as Foveabox, Feature Fusion SSD (FSSD), Task-Oriented Object Detection (TOOD), Efficientdet, YOLOv4, among others [14–24]. Through the analysis of performance variations among these models, the superiority of the model based on the stepwise fusion of residual attention and forward features is validated.

### Dataset

The dataset used in this study comprises a total of 597 images, with 478 images allocated for training and 119 images for testing. In the summer of 2017, four surgical videos were collected from the Department of Urology in Zhongnan Hospital of Wuhan University for the treatment of prostate hyperplasia, and were labeled by the doctors of the Department of Urology in Zhongnan Hospital of Wuhan University. Medical images present unique challenges compared to other datasets, particularly in terms of shape and contour determination. The prostate capsule is not an independent tissue but rather a layer of external capsule attached to the prostate. It is composed of collagen, smooth muscle, and striated muscle (the external urethral sphincter of the prostate capsule), which envelops and blends with the fibromuscular stroma of the prostate parenchyma. It is characterized by hash fibers, significant deformation, and non-uniform thickness. During examination, the outer capsule may resemble a white fatty tissue sheet on the prostate, making it difficult for untrained individuals to distinguish. Only trained personnel or experienced medical professionals can accurately judge the prostate capsule.

### Experimental environment

The deep learning networks in this study were trained using the Caffe and Pytorch frameworks. The hardware environment for Caffe consists of an Intel Core-i7-8700 CPU running at 3.2 GHz, 16 GB of memory, NVIDIA GTX 1070 or NVIDIA RTX 2060 graphics card, and Ubuntu Linux 64-bit operating system. The learning rate used in the Caffe environment was set to 0.0001. On the other hand, the Pytorch framework was utilized in a hardware environment with a 12 vCPU Intel® Xeon® E5-2650 v4 processor clocked at 2.20 GHz and a Tesla V100 graphics card with 32 GB of memory.

we will present experimental results from five key perspectives: comparing mAP and loss training curves, visualizing features, assessing speed and precision, conducting ablation experiments, and analyzing detection results.

### The mAP/loss curve

**Fig. 8:**
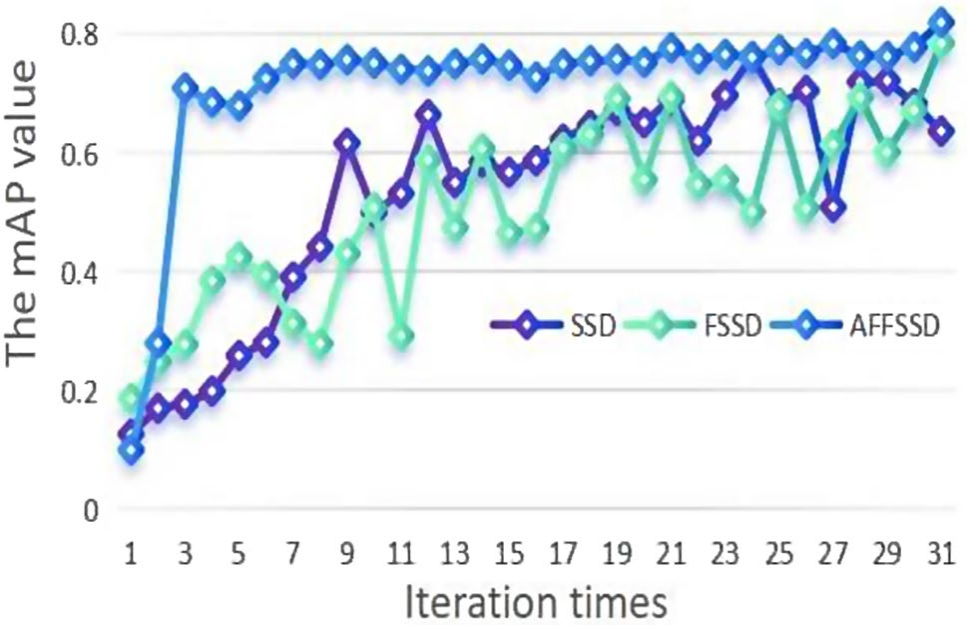
The mAP curve transformation (SSD, FSSD, AFFSSD)

The mAP curve evolution during training for SSD, FSSD, and AFFSSD models is depicted in Fig. 8, covering the initial 3100 training iterations.

**Fig. 9:**
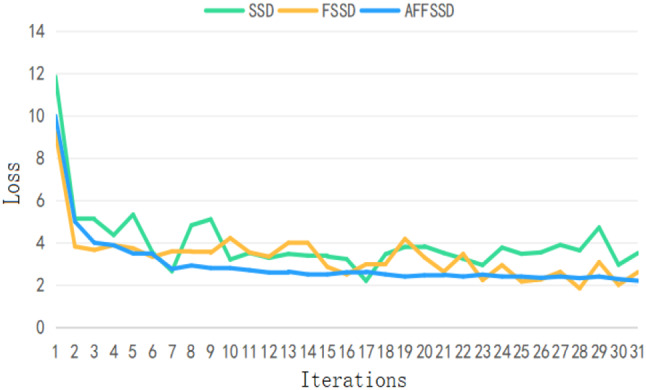
The Loss curve transformation comparison

The rapid improvement in mAP for AFFSSD is attributed to the integration of attention and feature fusion mechanisms. Meanwhile, FSSD exhibits significant fluctuations in mAP under the full training sample mode. The evolution of the loss curve during training is illustrated in Fig. 9, capturing the first 3100 training iterations.

Initially, the loss for AFFSSD was relatively high; however, it decreased rapidly, reaching approximately 2.5 after 700 iterations. With the progression of iterations, the loss for AFFSSD decreases at a faster rate and to a lower level compared to SSD and FSSD.

### Feature visualization

During the training of AFFSSD, SimAM was employed to boost the attention of convolution layers conv2_2, conv3_3, conv4_3 and cov5_3.

**Fig. 10:**
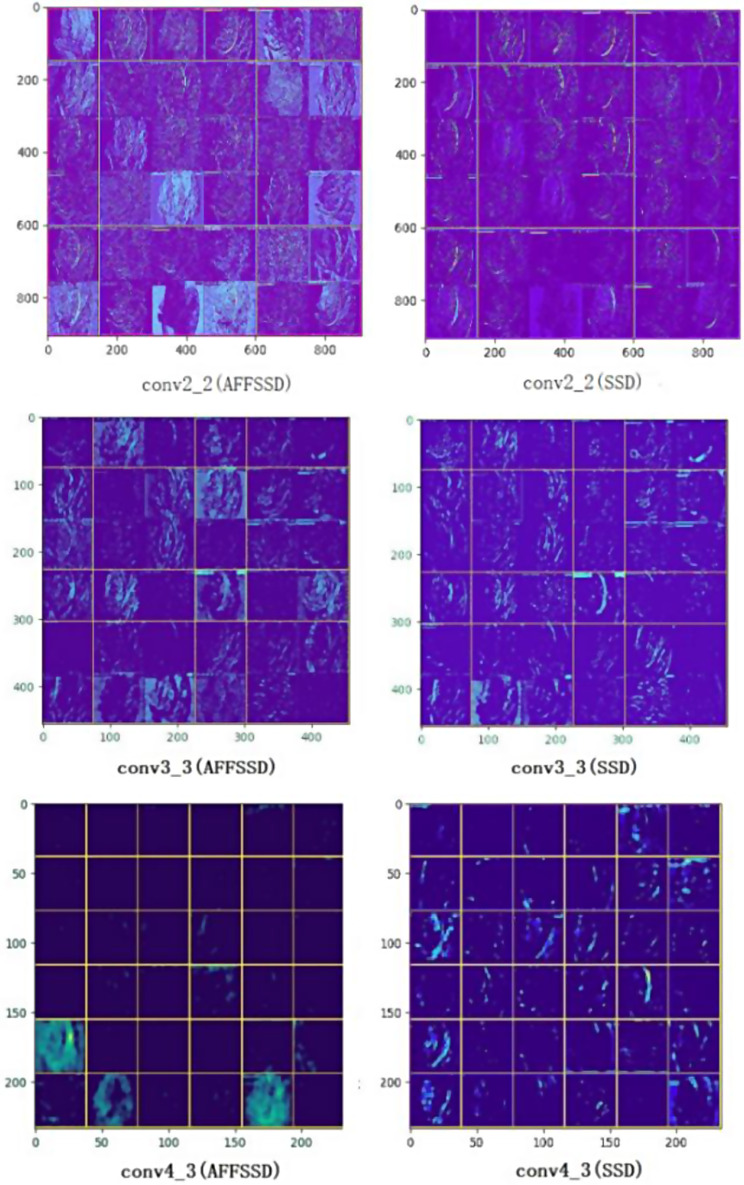
The feature visualization comparison(AFFSSD and SSD)

Following the attention enhancement by SimAM, the extracted features from the convolutional layer feature maps become more enriched. Typically, lower convolutions are responsible for localization, and the heightened attention to these lower convolutions aids in extracting decision-making features. Fig. 10 presents a visual comparison of features extracted from conv2_2, conv3_3, and conv4_3, showcasing the impact of attention enhancement.

### The speed and precision comparison

The proposed model achieves a speed of 0.014 ms on NVIDIA RTX 2060, making it suitable for real-time detection. Various methods, such as Faster R-CNN (ZF, VGG16, ResNet 50), SSD (VGG16, ResNet 101), EfficientDet (D0-D7), FoveaBox, TOOD, YOLOv4, Sparse R-CNN, Object Detection in Aerial Images without Object-level Supervision (OWOD), R-FCN (ResNet-50), and FSSD (VGG16), are compared in Table 1, which presents the settings and results of AFFSSD (VGG16-simAM) parameters.

**Table 1:** The Speed and precision comparison of various methods

Methods	Backbone network	mAP	FPS
Faster R-CNN [21]	ZF	62.00%	15
Faster R-CNN [21]	VGG16	62.67%	5 (K40)
Faster R-CNN [21]	ResNet 50 + FPN	74.41%	—
SSD [20]	VGG16	71.60%	46 (NVIDIA GTX 1070)
SSD [20]	ResNet-101 [165]	74.39%	15 (NVIDIA GTX 1070)
EfficientDet-D0 [23]	EfficientNets	53.38%	97 (Telsa v100)
EfficientDet-D1 [23]	EfficientNets	56.58%	74 (Telsa v100)
EfficientDet-D2 [23]	EfficientNets	59.23%	57 (Telsa v100)
EfficientDet-D3 [23]	EfficientNets	61.14%	35 (Telsa v100)
EfficientDet-D4 [23]	EfficientNets	58.81%	23 (Telsa v100)
EfficientDet-D5 [23]	EfficientNets	58.09%	10 (Telsa v100)
EfficientDet-D7 [23]	EfficientNets	77.21%	—
FoveaBox [18]	ResNet50 + FPN	81.10%	25 (NVIDIA RTX 2060)
TOOD [16]	ResNet50 + FPN	73.08%	20 (NVIDIA RTX 2060)
YOLOv4 [14]	CSPDarknet-53	70.29%	45 (NVIDIA RTX 2060)
YOLOv7 [24]	EfficientNet	74.20%	161 (Telsa v100)
Sparse R-CNN [22]	ResNet50 + FPN	75.68%	17 (NVIDIA RTX 2060)
OWOD [17]	ResNet-50	71.30%	62 (NVIDIA RTX 2060)
R-FCN [15]	ResNet 50	65.38%	12 (NVIDIA GTX 1070)
FSSD [19]	VGG16	73.50%	65.8 (NVIDIA GTX 1080Ti)
AFFSSD [ours]	VGG16-simAM	83.12%	72 (NVIDIA RTX 2060)

The model AFFSSD, which combines nonparametric attention fusion and progressive fusion of forward features, achieves a detection precision of 83.12%.

### The ablation experiment

**Table 2:** The improvement of different attention mechanism methods for SSD networks

ModelBackbone network	Attention network	Backbone network parameters	Attention network parameters	mAP
SSD(ResNet-50)	SENet [25]	24.37 M	2.514 M	73.27%
SSD(ResNet-50)	CBAM [26]	24.37 M	2.532 M	79.25%
SSD(ResNet-50)	SimAM [27]	24.37 M	0	75.57%
SSD(ResNet-50)	ECANet [28]	24.37 M	0	75.86%
SSD(ResNet-50)	Triplet [29]	24.37 M	0.005 M	75.39%
SSD(ResNet-50)	Split-Attention [30]	24.37 M	3.130 M	75.42%
SSD(VGG16)	SENet [25]	23.06 M	2.514 M	75.14%
SSD(VGG16)	CBAM [26]	23.06 M	2.532 M	76.31%
SSD(VGG16)	ECANet [28]	23.06 M	0	76.37%
SSD(VGG16)	SimAM [27]	23.06 M	0	79.26%
SSD(VGG16)	SANet [31]	23.06 M	0.005 M	74.40%

Before adopting SimAM, Table 2 compared the improvement results of various attention mechanism methods on SSD networks and selected the SimAM attention mechanism based on precision and number of parameters. Within the VGG16 framework, SimAM demonstrated the best performance and achieved the highest mAP when integrated with SSD.

**Table 3:** Residual attention fusion ablation experiment based on ASSD

Model	Backbone network	conv2_2	conv3_3	conv4_3	conv5_3	mAP
ASSD	VGG16-SimAM	✓	✓	✓	✓	79.26%
ASSD	VGG16-SimAM		✓	✓	✓	80.62%
ASSD	VGG16-SimAM	✓		✓	✓	75.54%
ASSD	VGG16-SimAM	✓	✓		✓	81.03%
ASSD	VGG16-SimAM	✓	✓	✓		77.37%
ASSD	VGG16-SimAM			✓	✓	75.91%
ASSD	VGG16-SimAM		✓		✓	80.01%
ASSD	VGG16-SimAM		✓	✓		75.59%
ASSD	VGG16-SimAM	✓			✓	82.19%
ASSD	VGG16-SimAM	✓		✓		76.05%
ASSD	VGG16-SimAM	✓	✓			74.91%
ASSD	VGG16-SimAM	✓				76.06%
ASSD	VGG16-SimAM		✓			74.26%
ASSD	VGG16-SimAM			✓		76.94%

In Table 3, different low-level convolutional combinations are compared, and the impact of residual attention fusion on the detection precision of SSD networks is discussed. When conv2_2 and conv5__3 both undergo residual attention fusion, the detection precision of ASSD can reach 82.19%. However, it is important to note that this combination scheme (conv2_2, conv5__3) does not necessarily work best when combined with the optimal combination of MFFSSD (conv2_2, conv3_3, conv4_3, fc7, conv6__2). The combined mAP achieved with this combination is only 80.04%. Table 4 presents the variation in detection precision of the object detection network with different feature fusion schemes. The forward feature stepwise fusion module that demonstrated the best performance in MFFSSD was selected based on mAP. This module includes four feature fusion modules formed by the stepwise fusion of conv2_2, conv3_3, conv4_3, fc7, and conv6_2.

**Table 4:** Progressive fusion module of forward feature based on MFFSSD

Model	Backbone network	conv2_2	conv3_3	conv4_3	fc7	conv6_2	mAP
MFFSSD	VGG16	✓	✓	✓	✓	✓	80.49%
MFFSSD	VGG16		✓	✓	✓	✓	76.13%
MFFSSD	VGG16	✓		✓	✓		79.18%
MFFSSD	VGG16	✓	✓	✓	✓		76.97%
MFFSSD	VGG16			✓	✓	✓	79.35%
MFFSSD	VGG16				✓	✓	80.86%
MFFSSD	VGG16		✓	✓	✓		77.76%
MFFSSD	VGG16	✓		✓			79.54%
MFFSSD	VGG16		✓	✓			80.47%
MFFSSD	VGG16			✓	✓		79.54%
MFFSSD	VGG16	✓	✓	✓			80.37%

**Table 5:** Ablation experiments based on AFFSSD

Model	Backbone network	conv2_2	conv3_3	conv4_3	conv5_3	mAP
AFFSSD	VGG16-SimAM-F	✓	✓	✓	✓	83.12%
AFFSSD	VGG16-SimAM-F		✓	✓	✓	78.47%
AFFSSD	VGG16-SimAM-F	✓		✓	✓	81.46%
AFFSSD	VGG16-SimAM-F	✓	✓		✓	78.98%
AFFSSD	VGG16-SimAM-F	✓	✓	✓		77.89%
AFFSSD	VGG16-SimAM-F			✓	✓	79.96%
AFFSSD	VGG16-SimAM-F		✓		✓	77.21%
AFFSSD	VGG16-SimAM-F		✓	✓		81.97%
AFFSSD	VGG16-SimAM-F	✓			✓	80.04%
AFFSSD	VGG16-SimAM-F	✓		✓		75.05%
AFFSSD	VGG16-SimAM-F	✓	✓			81.96%
AFFSSD	VGG16-SimAM-F			✓	✓	81.97%
AFFSSD	VGG16-SimAM-F	✓				79.91%
AFFSSD	VGG16-SimAM-F		✓			78.42%
AFFSSD	VGG16-SimAM-F			✓		79.63%
AFFSSD	VGG16-SimAM-F				✓	82.87%

In the progressive fusion experiments for forward features, Table 5 presents the mAP comparison results across different feature fusion strategies incorporating residual attention fusion. Vgg16-simam-F denotes an enhanced parameterless residual attention feature fusion and addressing resolution loss caused by downsampling. Additionally, the AFFSSD network utilizes a forward feature fusion approach to dynamically compensate for four-level semantic information.

**Fig. 11:**
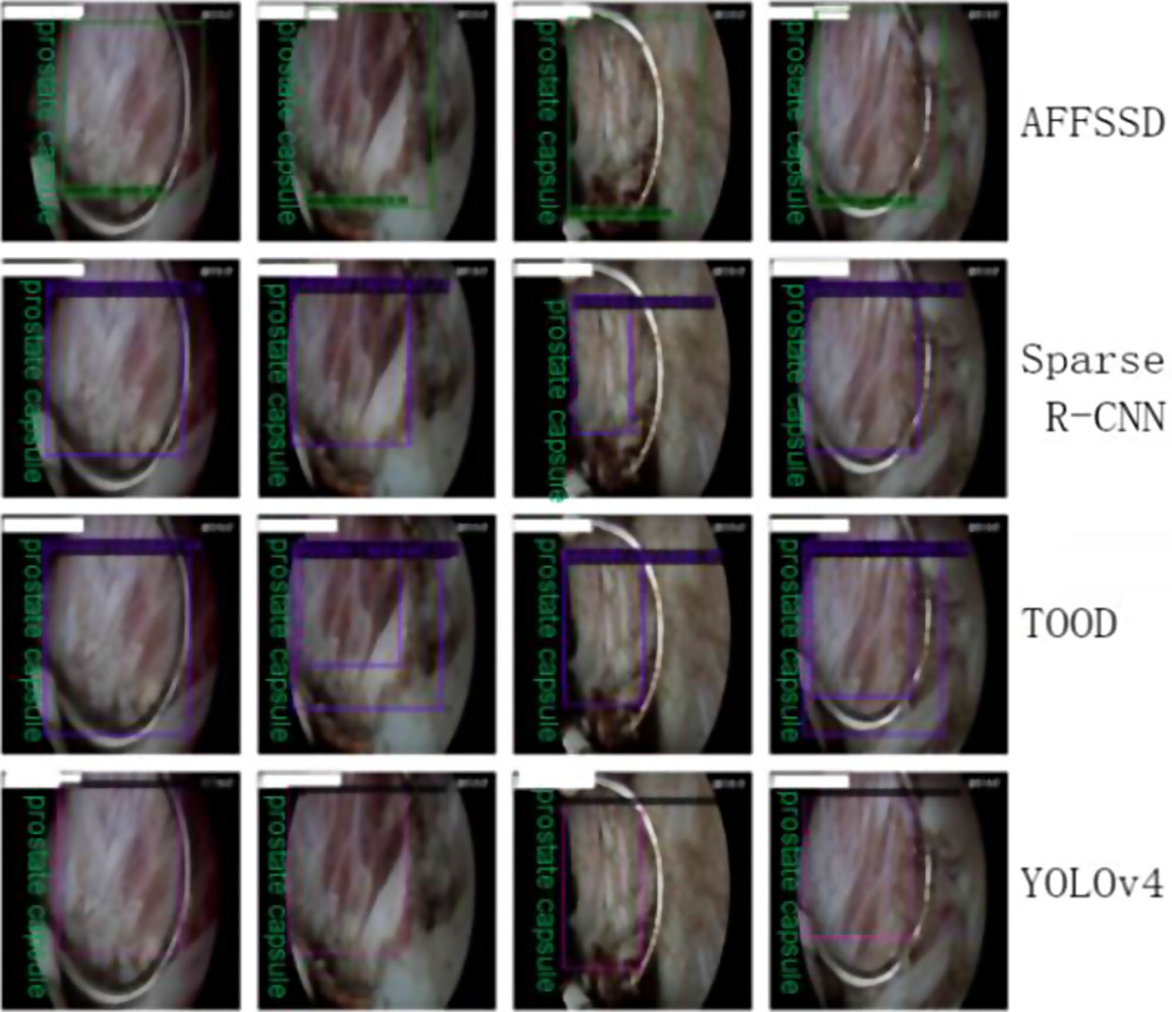
Detection result comparison

Fig. 11 displays the comparison of detection results between AFFSSD, TOOD, Sparse R-CNN, and YOLOv4. The performance of the comparison networks is limited, possibly due to training results that do not converge effectively with the small dataset. AFFSSD achieved superior detection results by incorporating the SimAM attention enhancement mechanism for texture-related convolutions like conv2_2 and conv3_3, utilizing parameterless residual attention feature fusion for lower-level features, and addressing resolution loss from downsampling. The AFFSSD network employs a forward feature fusion method to progressively integrate four-level semantic information for improved performance.

## Discussion

The limited dataset may have hindered the training convergence of the comparison networks, leading to their underdeveloped performance. In contrast, AFFSSD achieves superior detection results by leveraging the SimAM attention enhancement mechanism for texture-related convolutions, such as conv2_2 and conv3_3. This is achieved through parameterless residual attention feature fusion that enhances lower-level features. Furthermore, AFFSSD addresses resolution loss caused by downsampling, contributing to its improved detection performance.

Utilizing the compact VGGNet enables the model to achieve high precision with reduced computational complexity, thereby accelerating inference speed. The non-parametric attention residual fusion method enhances the network’s representation learning capability, minimizing redundant information and boosting inference speed. Furthermore, employing multi-scale detection techniques enhances the network’s detection performance and speed across various scales.

Our experiments have shown that a small model on a small dataset yields better results. Using a large model on a small dataset can lead to overfitting, as the model may memorize the samples rather than learn their general features. In contrast, small models are simpler and more likely to generalize to new samples outside the small dataset. Small models require fewer parameters and computational resources, making them suitable for training on small datasets. This allows for faster model training, avoiding overfitting and resource wastage. Small models focus more on learning key features of the data, avoiding the confusion of noise and irrelevant features that overly complex models may encounter. They are typically easier to interpret and understand, enabling better insights into patterns and trends within the data.

Therefore, training with a small model on a small dataset can effectively utilize the data, mitigate overfitting, and deliver better performance in practical applications.

One major limitation of this study is the small dataset. It is recommended to utilize a combination of generative adversarial networks and deep active learning methods to augment the dataset in the future.

## Conclusions

The proposed model demonstrates impressive speed capabilities, achieving an inference time of 0.014 ms on an NVIDIA RTX 2060, enabling rapid detection. The AFFSSD model, which comprises unparametric attention fusion and progressive fusion of forward features, achieves a high detection precision of 83.12%. When compared to popular object detection models such as Faster R-CNN (ZF, VGG16, ResNet 50), SSD (VGG16, ResNet 101), EfficientDet (D0-D7), FoveaBox, TOOD, YOLOv4, Sparse R-CNN, OWOD, R-FCN (ResNet-50), and FSSD (VGG16), the proposed AFFSSD method outperforms them with the highest mean Average Precision (mAP) while maintaining faster speeds, only slightly slower than YOLOv7.

This paper enhances the inference capability, detection speed, and detection precision of the object detection network using methods such as parameter-free attention, residual fusion, and progressive feature propagation. Achieving a balance between speed and precision using a small model on a small dataset.

This article focuses on discussing real-time detection of medical images. Real-time detection can help doctors accurately locate lesion areas or target tissues, thereby avoiding damage to healthy tissues or organs. Through real-time detection, doctors can promptly identify and address unexpected situations or complications that may arise during surgery, improving the safety and success rate of the procedure. Real-time detection results can provide doctors with timely feedback, assisting them in making adjustments and decisions to ensure the smooth progress of the surgery. The location of the detected prostate capsule is marked with a box on the display screen, and a buzzer sounds to alert the doctor. After viewing the real-time test results, the doctor finally determines whether the algorithm has flagged the prostate capsule. Accurate real-time detection results may impact post-operative treatment outcomes and patient recovery, thereby enhancing the therapeutic effect and prognosis of the surgery.

## Data Availability

The datasets used and/or analysed during the current study are available from the corresponding author on reasonable request.

## Abbreviations

SimAM: Simple, Parameter-Free Attention Module
Faster R-CNN: Faster Region-based Convolutional Neural Network
SSD: Single Shot Multibox Detector
AFFSSD: Attention based Feature Fusion Single Shot Multibox Detector
mAP: Mean average precision
YOLOv7: You Only Look Once version 7
3DMedPT: 3D Medical Point Converter
AIS: acute ischemic stroke
CT: Computed Tomography
GS: Gleason score
MRI: Magnetic Resonance Imaging
CNN: Convolutional Neural Network
ASSD: Attention-based single shot multibox
ReLU: Rectified Linear Units
MFFSSD: Multi-scale Feature Fusion Single Shot Multibox Detector
R-FCN: Region-based Fully Convolutional Networks
FSSD: Feature Fusion SSD
TOOD: Task-Oriented Object Detection
OWOD: Object Detection in Aerial Images without Object-level Supervision
FPS: Frame Per Second
